# Development and validation of a transformer model-based early warning score for real-time prediction of adverse outcomes in the emergency department

**DOI:** 10.1038/s41598-025-07511-7

**Published:** 2025-07-02

**Authors:** Hansol Chang, Jong Eun Park, Daehwan Lee, Kiwon Lee, Se Yong Jekal, Ki Tae Moon, Sejin Heo, Doyeop Kim, Gun Tak Lee, Sung Yeon Hwang, Won Chul Cha, Wonhee Kim, Tae Ho Lim, Tae Gun Shin

**Affiliations:** 1 Department of Emergency Medicine, Samsung Medical Center, Sungkyunkwan University School of Medicine, 115 Irwon-ro Gangnam-gu, Seoul, 06355 Republic of Korea; 2 Department of Digital Health, Samsung Advanced Institute for Health Science & Technology, Sungkyunkwan University, 115 Irwon-ro Gangnam- gu, Seoul, 06355 Republic of Korea; 3 Spidercore Inc, Daehakro 99, Yuseong-gu, Daejeon, Republic of Korea; 4 Data Service Team, Samsung Medical Center, 81 Irwon-ro Gangnam-gu, Seoul, 06351 Republic of Korea; 5 Department of Biomedical Informatics, Ajou University School of Medicine, Suwon, Republic of Korea; 6 Department of Emergency Medicine, Kangnam Sacred Heart Hospital, Hallym University College of Medicine, Seoul, Republic of Korea; 7 Department of Emergency Medicine, Hanyang University College of Medicine, Seoul, 04763 Republic of Korea

**Keywords:** Early medical intervention, Critical care, Emergency department, Machine learning, Outcomes research, Hypoxia

## Abstract

This study aimed to develop and validate a transformer-based early warning score (TEWS) system for predicting adverse events (AEs) in the emergency department (ED). We conducted a retrospective study analyzing adult ED visits at a tertiary hospital. The TEWS was developed to predict five AEs within 24 h: vasopressor use, respiratory support, intensive care unit admission, septic shock, and cardiac arrest. Performance was evaluated and compared using the area under the receiver operating characteristic curve (AUROC) and bootstrap-based t-test. External validation was performed using the Marketplace for Medical Information in Intensive Care (MIMIC)-IV-ED database. Transfer learning was applied using 1% and 5% of the external data. A total of 414,748 patients was analyzed in the development cohort (AEs, 3.7%), and 410,880 patients (AEs, 6.7%) were included in the external validation cohort. Compared to the modified early warning score (MEWS), the TEWS incorporating 13 variables and the vital signs-only TEWS demonstrated superior prognostic performance across all AEs. The AUROC ranged from 0.833 to 0.936 for TEWS and 0.688 to 0.874 for MEWS. In external validation, the TEWS also showed acceptable discrimination with AUROC values of 0.759 to 0.905. Transfer learning significantly improved the performance, increasing AUROC values to 0.846–0.911. The TEWS system was successfully integrated into the electronic health record (EHR) system of the study hospital, providing real-time risk assessment for ED patients. We developed and validated an artificial intelligence-based early warning score system that predicts multiple adverse outcomes in the ED and was successfully integrated into the EHR system.

**Supplementary Information:**

The online version contains supplementary material available at 10.1038/s41598-025-07511-7.

## Introduction

Effectively screening patients at risk of clinical deterioration is crucial in the emergency department (ED) to ensure timely intervention and improve patient outcomes^1^. Limited resources and space, combined with an uncontrolled influx of patients, present significant challenges that require rigorous management. Thus, prioritizing patients needing immediate care, efficiently allocating resources, and implementing appropriate interventions are essential to providing optimal patient care^2–4^.

Early warning systems (EWS) such as the Modified Early Warning Score (MEWS) and National Early Warning Score (NEWS) have been widely used in EDs to identify patients at risk of clinical deterioration^5,6^. These tools are easy to use and facilitate rapid risk stratification, but their accuracy and generalizability are limited by fixed scoring rules and reliance on a small set of variables. To potentially overcome the limitations of conventional early warning scores and enhance the screening of high-priority patients, various artificial intelligence (AI)-based clinical decision support systems (CDSS) have been introduced to support clinical decision-making and improve patient triage^7–11^. These systems assist in real-time clinical decision-making by primarily predicting patient outcomes, such as mortality rates or probability of intensive care unit (ICU) admission. However, a significant gap remains in AI-based CDSS: the ability to adaptively provide updated results as patient status changes over time and to specify necessary interventions. This is particularly important because the conditions of patients in the ED are dynamic and can change rapidly^7,8,10,12–14^.

Transformer learning is a novel approach that offers enhanced data processing capabilities, efficiently managing the large and complex datasets commonly encountered in medical settings^15^. Transformers are ideal for CDSS in EDs due to their ability to handle irregular time series data, adapt continuously, manage missing data, and offer interpretability^16–18^. Using time embeddings and attention mechanisms, transformers capture important patterns across uneven time intervals, which are common in ED data^19,20^. Transformers support continuous re-learning, allowing the CDSS to remain current based on emerging clinical knowledge^21^. With attention-based weighting, transformers can address missing data effectively by focusing on the most informative inputs^22^. Furthermore, the model’s attention weights offer interpretability, allowing clinicians to visualize the factors that influence the decision, enhancing transparency and trust^23^.

This study aims to develop and validate a transformer model-based early warning score (TEWS) that can reflect real-time changes in patient status and provide specific intervention recommendations. Furthermore, we sought to develop a system that integrates the TEWS into the electronic health record (EHR) system to provide clinical decision support and actionable information to healthcare providers.

## Methods

### Study setting and population

The study was retrospective and observational, using data from patient ED visits at one tertiary referral hospital in Republic of Korea with approximately 1,980 inpatient beds and approximately 60,000 ED visits per year. Adult patients (aged 19 years or older) who visited the ED of the study site between 2015 and 2022 were included in this study. We excluded patients who had signed “Do not attempt resuscitation” orders and those whose vital signs were not measured in the ED.

For external validation, the Marketplace for Medical Information in Intensive Care (MIMIC)-IV-ED database was used. Patients (aged 19 years or older) with data between 2011 and 2019 were included^24^. This study was conducted in accordance with the TRIPOD-AI (Transparent Reporting of a multivariable prediction model for Individual Prognosis Or Diagnosis-Artificial Intelligence) guidelines^25^.

### Prediction outcome

The purpose of the predictive model was to estimate the likelihood of the five adverse events (AEs) of critical interventions and outcomes occurring within 24 h; (1) the use of vasopressors (norepinephrine, epinephrine, dopamine, and vasopressin infusion), (2) advanced respiratory support (high-flow nasal cannula, noninvasive positive ventilation, and mechanical ventilation intubation), (3) ICU admission, (4) progression to septic shock (according to the Sepsis-3 definition)^26^, and (5) in-hospital cardiac arrest.

### Data preprocessing

Data were extracted from the hospital’s clinical data warehouse, including demographic characteristics, vital signs, laboratory test results, procedural events, drug administration, and outcomes. Preprocessing proceeded sequentially through the following steps: (1) outlier detection and removal, (2) normalization, (3) resampling and windowing, and (4) data handling. Outliers were identified and excluded based on clinically acceptable ranges for vital signs and reportable ranges for laboratory data. (Table S1). All variables were normalized to a 0–1 range using min-max scaling. The normalized data were combined into multi-dimensional vectors according to the number of variables and into two-dimensional vectors for age and gender.

To reflect the temporal changes in variables, the data were structured as time series composed of 15-minute intervals over 48 h. The 48-hour observation window was implemented as a sliding window aligned with each prediction time point, incorporating data from the most recent 48 h. For most patients who stayed in the ED for less than 48 h after arrival, only the available data were used as model input. If multiple data points existed within a 15-minute interval, the last data point was used, and missing data points were replaced with ‘0.’ This resulted in data with a (192, N) format. Labeling was designed to capture the timing of AEs. Label 1 (acute deterioration) was assigned if AEs occurred within 24 h from the last timestamp, while Label 0 (normal) was assigned if no acute deterioration occurred within 24 h. The dataset was split into training, validation, and test sets in a 60%, 20%, and 20% ratio based on patient admission numbers.

After finalizing a data use agreement and completing the “Protection of Human Subjects” training, researchers can access the MIMIC-IV database online (https://mimic.physionet.org). We obtained authorization to access the MIMIC-IV database. Researchers who completed the Collaborative Institutional Training Initiative (CIIT) program extracted the following data using PostgreSQL tools version 15.4 (PostgreSQL Global Development Group, Berkeley, CA, USA) and Python version 3.7.5. We obtained authorization and accessed the MIMIC-IV database on 27 October 2021. The data codes used for the study analyses were from the MIMIC Code Repository^27^.

### Model and training

**Fig. 1:**
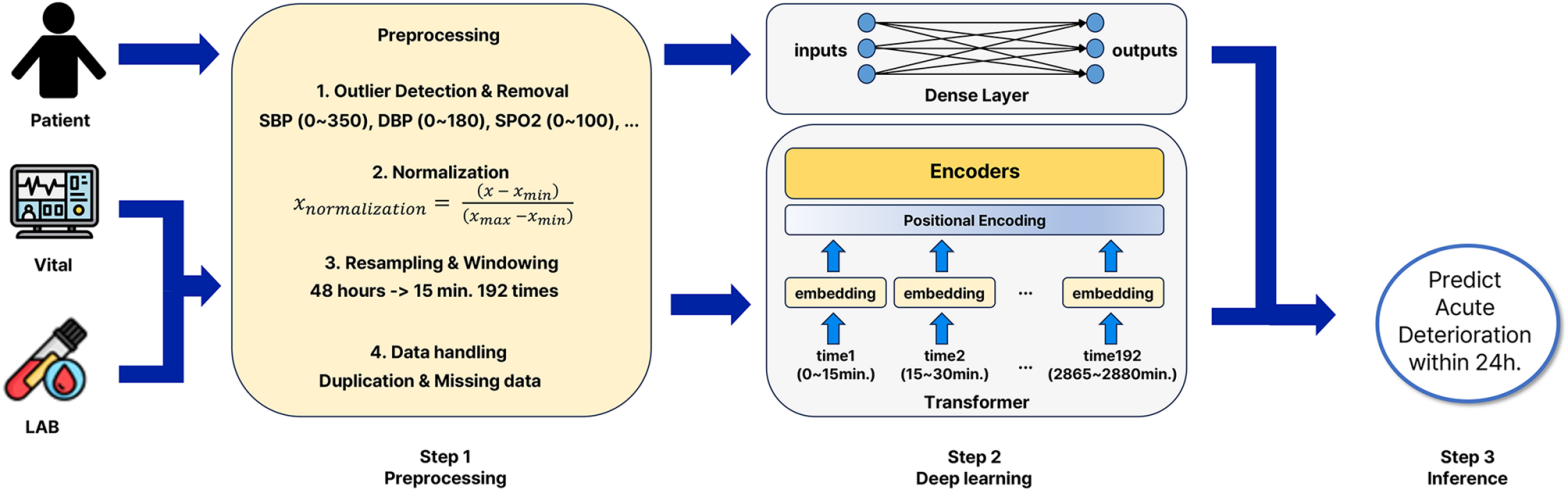
Overview of the Transformer-Based Early Warning Score model.

We developed and tested separate transformer models for five adverse outcomes (Fig. 1).

The input to the TEWS model consists of two components: a multivariate time-series sequence (after preprocessing) and a set of static features. For each patient $\:i$, the time-series input is denoted as:

$$\:{X}_{i}={\left\{{\mathbf{x}}_{i}^{\left(t\right)}\right\}}_{t=1}^{T},\hspace{1em}{\mathbf{x}}_{i}^{\left(t\right)}\in\:{R}^{d}$$

where $\:{\mathbf{x}}_{i}^{\left(t\right)}$ represents the observed features (e.g., vital signs and laboratory results) at time step t, sampled every 15 min over a 48-hour window, resulting in $\:T=192$ steps. In addition, static patient-level information such as age, sex, and mode of arrival is encoded as:

$$\:{\varvec{s}}_{i}\in\:{R}^{k}$$

The final model input is the combination of both modalities:

$$\:{x}_{i}=\left({X}_{i},\:{\varvec{s}}_{i}\right)$$

The time-series component $\:{X}_{i}$​ is processed by a 4-layer Transformer encoder with single-head self-attention and a hidden dimension of 512. To preserve temporal ordering, standard sinusoidal positional encoding is added to the input sequence prior to encoding.

The static vector $\:{\varvec{s}}_{i}$​ is passed through a fully connected (FC) layer. The resulting representations are concatenated and passed through a sigmoid activation to produce the predicted probability of an adverse event:

$$\:\widehat{{y}_{i}}={\upsigma\:}(\mathbf{W}\:\cdot\:\left[\text{T}\text{r}\text{a}\text{n}\text{s}\text{f}\text{o}\text{r}\text{m}\text{e}\text{r}\left({X}_{i}\right),\left.\vert\vert \text{F}\text{C}\left({\varvec{s}}_{i}\right)\right]+b\right)$$

where $\:\Vert\:$ denotes vector concatenation, $\:\sigma\:$ is the sigmoid activation function, and $\:\mathbf{W},b$ are the learnable weight matrix and bias term, respectively.

During training, the binary cross-entropy loss function was used. To address the issue of data imbalance, higher weights were assigned to cases of acute deterioration. The loss function was defined as follows:

$$\:{L}_{BCE}=-\frac{1}{N}{\sum\:}_{i=1}^{N}\left[{\omega\:}_{pos}\cdot\:{y}_{i}\text{log}\left(y\hat {}_{i}\right)+{\omega\:}_{neg}\cdot\:\left(1-{y}_{i}\right)\text{log}\left(1-y\hat {}_{i}\right)\right]$$

$$\:\left({\omega\:}_{pos}=5.495,\:{\omega\:}_{neg}=0.505\right)$$

where N represents the number of samples, $\:{y}_{i}\in\:\left\{\text{0,1}\right\}$ represents the actual label of the ith sample, $\:y\hat {}_{i}$ represents the model’s predicted probability for the ith sample, $\:{\omega\:}_{pos}$ represents the weight for the positive class (Label 1), and $\:{\omega\:}_{neg}$ represents the weight for the negative class (Label 0). The weights were calculated using scikit-learn’s compute_class_weight function. Adjusting the weights of the loss function enhanced the model sensitivity to relapse.

We optimized the hyperparameters using a grid search, training the TEWS model with all possible combinations. Each configuration was evaluated based on the validation loss, and the combination that yielded the lowest loss was selected as the best.

The learning rate was set at 1e-4, with AdamW used as the optimizer and a batch size of 1000. The model was trained for a total of 200 epochs. The entire code was written using Python v3.7.5, and the machine learning algorithm was implemented using TensorFlow v2.5.0. The GPU used was TITAN V, with CUDA version 11.2 and cuDNN version 8.9.1.

### Model explainability

To ensure that the TEWS model is interpretable and actionable in clinical settings, we incorporated model explainability using gradient-weighted class activation mapping (Grad-CAM)^28^. Grad-CAM, originally designed for convolutional neural networks, was adapted to analyze the importance of features in our transformer model. By visualizing the model’s attention to different features, Grad-CAM allowed us to identify the variables contributing most highly to the predictions of the model at specific time intervals for individual cases.

Through this analysis, we observed that the top contributing features varied across individual patients, reflecting the ability of the model to dynamically adapt to unique clinical presentations. This case-specific explainability was integrated into the EHR system, allowing clinicians to view the most relevant features influencing the predictions of the model for each patient in real time^9^.

### Feature selection

We tested 44 variables and conducted multiple-step model testing with feature reduction to select the optimal features for our models (Table S1). The key consideration in the feature selection process was to ensure acceptable prognostic performance^29^. Features were selected based on their high contribution to model performance and their consistent appearance across models. The final feature selection also considered practical aspects such as data processing efficiency, number of measurements, computational costs, ease of EMR integration, and feasibility of external validation. We tested a full TEWS model with selected features and a TEWS model using vital signs only.

### Model performance measure and validation

We measured and compared the model performance by area under the receiver operating characteristic curve (AUROC) for predicting each outcome. Additionally, we calculated the area under the precision-recall curve (AUPRC), sensitivity, specificity, positive predictive value (PPV), and negative predictive value (NPV) at the optimal cut-off determined by the Youden Index. The TEWS model output was displayed as a score ranging from 0 to 1. Each score was categorized into three risk groups (low, intermediate, and high risk) using cut-off values selected to achieve sensitivity of 90% and specificity of 95%. These initial thresholds were further refined for each outcome by considering false alarm counts, PPV, and NPV to optimize clinical implementation.

We compared the performance of our TEWS model with the MEWS^30^, calculated at the same time intervals. Setting the MEWS threshold at ≥ 5 points as the reference, we evaluated the performance of TEWS at a comparable sensitivity level and calculated the number of false alarms per 1,000 patients^31^.

For external validation, we evaluated the predictive performance of the TEWS models for each outcome using the MIMIC-IV-ED database. The cardiac arrest prediction model was not evaluated as cardiac arrest occurrence data are not available in the MIMIC-IV-ED database.

To improve model performance during external validation, we used transfer learning by fine-tuning the model with 1% (approximately 1,600 patients) and 5% of the MIMIC-IV-ED database^32^. This enabled the model to adapt to the characteristics of the external dataset. Performance evaluation was conducted using an independent 20% subset of the MIMIC-IV-ED database. In addition, we tested the performance of alternative models including logistic regression and XgBoost models using the final variables.

### Statistical analysis

Categorical variables are reported as frequencies and percentages, while continuous variables are reported as means (standard deviations, SD). The significance of differences in continuous variables between groups was assessed using Student’s t-test, while differences in categorical variables between groups were analyzed using chi-square tests. Confidence intervals were calculated using bootstrapping. To assess the statistical significance of the differences in diagnostic performance including AUROC between TEWS and MEWS, we performed a bootstrap-based t-test with 1,000 resamples. A two-tailed p value < 0.05 was considered statistically significant. All analyses were performed using R version 3.6.3. (R Foundation for Statistical Computing, Vienna, Austria) and Python version 3.7.5.

## Results

### Demographics

**Fig. 2:**
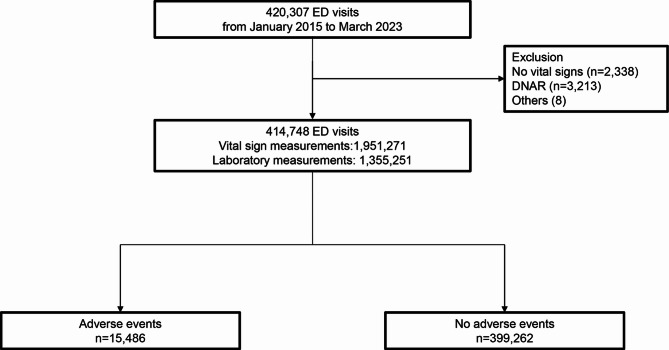
Study population.

A total of 414,748 subjects was analyzed, among whom 15,486 (3.7%) experienced AEs (Fig. 2). Baseline characteristics are shown in Table 1. The vital signs and laboratory variables were significantly worse in the group that experienced AEs compared to those without AE. AEs included vasopressor use (*n* = 6,304, 40.7%), respiratory support (*n* = 5,017, 32.4%), ICU admission (*n* = 8,492, 54.9%), septic shock (*n* = 4,124, 26.6%), and cardiac arrest (*n* = 548, 3.5%). For external validation, 410,880 patients (27,838 AEs, 6.7%) were analyzed from the MIMIC-IV-ED database (Figure S1 and Table S2).

**Table 1:** Basic characteristics of the study population.

Variables	At least one AE (*n* = 15,486)	Without AE (*n* = 399,262)	*P*-value
Age, mean ± SD	65.0 ± 14.7	55.3 ± 17.7	< 0.001
Sex, n (%)			< 0.001
Male	5,944 (38.4%)	206,506 (51.7%)	
Female	9,542 (61.6%)	192,756 (47.3%)	
Vital signs (the first measurement)			
SBP (mmHg), mean ± SD	122.0 ± 34.8	132.4 ± 24.6	< 0.001
DBP (mmHg), mean ± SD	71.6 ± 21.3	79.8 ± 15.3	< 0.001
HR (beats/min), mean ± SD	101.3 ± 28.2	88.9 ± 19.3	< 0.001
RR (breaths/min), mean ± SD	21.3 ± 6.0	18.5 ± 3.2	< 0.001
Body Temperature (°C), mean ± SD	37.1 ± 1.2	36.9 ± 0.8	< 0.001
SpO_2_ (%), mean ± SD	93.9 ± 9.1	97.6 ± 4.3	< 0.001
Laboratory tests (the first measurement)			
Hb (g/dl), mean ± SD	11.4 ± 2.8	12.5 ± 2.4	< 0.001
BUN (mg/dl), mean ± SD	29.4 ± 23.9	18.0 ± 16.3	< 0.001
Sodium (mmol/L), mean ± SD	134.6 ± 6.9	137.7 ± 4.7	< 0.001
Potassium (mmol/L), mean ± SD	4.4 ± 1.2	4.2 ± 0.6	< 0.001
Lactate (mmol/L), mean ± SD	3.6 ± 3.8	1.7 ± 1.2	< 0.001
Arterial pH, mean ± SD	7.4 ± 0.2	7.4 ± 0.1	< 0.001
Arterial HCO_3_ (mmol/L), mean ± SD	20.9 ± 6.7	23.2 ± 4.5	< 0.001
MEWS (the first measurement), mean ± SD	3.2 ± 1.9	1.7 ± 1.1	< 0.001
KTAS, n (%)			< 0.001
1	1,543 (10.0%)	876 (0.2%)	
2	4,954 (32.0%)	20,585 (5.2%)	
3	7,252 (46.8%)	163,401 (40.9%)	
4	1,129 (7.3%)	152,011 (38.1%)	
5	66 (0.4%)	20,304 (5.1%)	
Not available	542 (3.5%)	42,085 (10.5%)	
Mode of arrival, n (%)			< 0.001
Ambulance	8,398 (54.2%)	70,517 (17.7%)	
Other	7,088 (45.8%)	328,745 (82.3%)	
ED disposition, n (%)			< 0.001
Discharge	379 (2.4%)	286,211 (71.7%)	
ED death	1,085 (7.0%)	432 (0.1%)	
Transfer	704 (4.5%)	12,286 (3.1%)	
Admission	13,318 (86.0%)	100,333 (25.1%)	
Adverse events, n (%)			< 0.001
Vasopressor use	6,304 (40.7%)	-	
Respiratory support	5,017 (32.4%)	-	
ICU admission	8,492 (54.9%)	-	
Septic shock	4,124 (26.6%)	-	
Cardiac arrest	548 (3.5%)	-	

*AE* adverse event, *SD* standard deviation, *SBP* systolic blood pressure, *DBP* diastolic blood pressure, *HR* heart rate, *RR* respiratory rate, *SpO*_2_ peripheral oxygen saturation, *Hb* hemoglobin, *BUN* blood urea nitrogen, *MEWS* modified early warning score, *KTAS* Korean Triage and Acuity Scale, *ED* emergency department, *ICU* intensive care unit.

### Model development and performance

The full TEWS model incorporated 13 key variables selected through iterative feature reduction from the initial 44 variables (Fig. S2). The model included vital signs (systolic blood pressure, diastolic blood pressure, heart rate, respiratory rate, body temperature, and peripheral oxygen saturation) and laboratory values (hemoglobin, blood urea nitrogen, sodium, potassium, lactate, arterial pH, and bicarbonate). The vital sign-only TEWS included the six vital signs mentioned above.

The TEWS (full model) demonstrated superior prognostic performance compared to MEWS across all adverse outcomes; vasopressor use: 0.934 (95% CI: 0.932–0.936), respiratory supports: 0.909 (95% CI: 0.905–0.912), ICU admission: 0.855 (95% CI: 0.853–0.856), septic shock: 0.936 (95% CI: 0.933–0.938), and cardiac arrest: 0.833 (95% CI: 0.820–0.848) (Table 2). Similarly, the vital sign-only TEWS model also exhibited better predictive performance than MEWS. Further results about the performance matrix and the other predictive models (transformer models with 23 and 44 variables, logistic regression, XgBoost) are shown in Tables S3, S4, and S5.

**Table 2:** Comparative prognostic performance of TEWS and MEWS models in predicting adverse outcomes.

	AUROC (95% CI), TEWS (full model)	AUROC (95% CI), Vital sign only TEWS	AUROC (95% CI), MEWS
Vasopressor use	0.934 (0.932–0.936)	0.928 (0.926–0.931)	0.825 (0.821–0.828)
Respiratory support	0.909 (0.905–0.912)	0.895 (0.891–0.899)	0.796 (0.790–0.801)
ICU admission	0.855 (0.853–0.856)	0.841 (0.838–0.844)	0.716 (0.712–0.720)
Septic shock	0.936 (0.933–0.938)	0.928 (0.924–0.931)	0.838 (0.833–0.843)
Cardiac arrest	0.833 (0.820–0.848)	0.825 (0.809–0.841)	0.782 (0.764-0.800)

*TEWS* transformer-based early warning score, *MEWS* modified early warning score, *AUROC* area under the receiver operating characteristic curve, *CI* confidence interval, *ICU* intensive care unit.

*P < 0.05 for all comparisons with MEWS.

When comparing TEWS and MEWS at similar sensitivity thresholds (MEWS ≥ 5), TEWS demonstrated superior performance with significantly higher specificity (99.0-99.5% vs. 96.0-96.8%), PPV (25.3–56.6% vs. 9.5–18.6%), and NPV (99.4–100.0% vs. 98.4–99.5%) across outcomes (Table 3). Additionally, TEWS showed substantially lower false alarm counts per 1,000 patients compared to MEWS.

**Table 3:** Comparison of accuracy and false alarm counts per 1,000 patients between TEWS and MEWS with similar sensitivity.

	Sensitivity, % (95% CI)	Specificity, % (95% CI)	PPV, % (95% CI)	NPV, % (95% CI)	False alarm counts per 1,000 patients (95% CI)
Vasopressor use					
MEWS ≥ 5	49.0 (46.6–51.5)	96.8 (96.7–96.9)	18.6 (17.5–19.8)	99.2 (99.2–99.3)	32 (30.4–32.6)
TEWS ≥ 0.983	49.6 (47.1–51.9)	99.5* (99.5–99.5)	56.6* (53.9–59.2)	99.3* (99.3–99.4)	5* (4.6–5.4)
Respiratory support					
MEWS ≥ 5	37.1 (34.4–40.0)	96.4 (96.3–96.5)	9.5 (8.6–10.4)	99.3 (99.3–99.4)	35 (34.3–36.5)
TEWS ≥ 0.975	38.5 (35.9–41.4)	99.0* (98.9–99.0)	25.3* (23.2–27.3)	99.4* (99.4–99.5)	10* (9.7–10.8)
ICU admission					
MEWS ≥ 5	29.1 (27.2–30.9)	96.5 (96.4–96.6)	15.3 (14.3–16.3)	98.4 (98.4–98.5)	34 (33.0-35.2)
TEWS ≥ 0.921	29.1 (27.3–31.0)	98.7* (98.6–98.8)	29.7* (27.9–31.6)	98.7* (98.6–98.7)	13* (12.2–13.4)
Septic shock					
MEWS ≥ 5	53.4 (50.5–56.4)	96.6 (96.5–96.7)	13.2 (12.3–14.2)	99.5 (99.5–99.6)	34 (32.5–34.6)
TEWS ≥ 0.976	54.3 (51.2–57.2)	99.3* (99.2–99.3)	38.0* (35.3–40.4)	99.6* (99.6–99.7)	7* (6.8–7.8)
Cardiac arrest					
MEWS ≥ 5	38.2 (29.7–46.6)	96.0 (95.9–96.1)	1.1 (0.8–1.4)	99.9 (99.9–99.9)	40 (38.6–41.0)
TEWS ≥ 0.872	38.9 (30.4–47.3)	97.8* (97.8–97.9)	1.8* (1.3–2.3)	100.0* (99.9–100.0)	22* (20.8–22.3)

TEWS, Transformer-Based Early Warning Score; MEWS, Modified Early Warning Score; PPV, Positive Predictive Value; NPV, Negative Predictive Value; CI, Confidence Interval.

*P < 0.05 compared with MEWS.

### External validation

In external validation using MIMIC-IV-ED data, TEWS demonstrated superior performance compared to MEWS across all outcomes **(**Table 4**).** Initially, the vital sign-only TEWS showed better performance than the full model, with AUROC values ranging from 0.815 to 0.905 compared to 0.759–0.872 for the full model. After applying transfer learning, both models showed significant improvement. With 1% data transfer learning, the performance of the full model improved (AUROC 0.851 to 0.903); with 5% data transfer learning, it improved further (AUROC 0.863 to 0.901). The full model generally outperformed the vital sign-only model after transfer learning.

**Table 4:** Area under the receiver operating characteristic of MIMIC-IV-ED data validation for predicting the adverse outcomes.

	TEWS (full model) (95% CI)	Vital sign only TEWS (95% CI)	MEWS
No transfer learning model			
Vasopressor use	0.847 (0.837–0.857)	0.882 (0.873–0.890)	0.828 (0.817–0.839)
Respiratory support	0.759 (0.753–0.763)	0.837 (0.833–0.841)	0.706 (0.699–0.713)
ICU admission	0.777 (0.774–0.780)	0.815 (0.812–0.819)	0.688 (0.684–0.692)
Septic shock	0.872 (0.857–0.889)	0.905 (0.891–0.918)	0.874 (0.857–0.887)
Transfer learning model (1% of data)			
Vasopressor use	0.870 (0.862–0.878)	0.889 (0.880–0.898)	-
Respiratory support	0.875 (0.871–0.879)	0.851 (0.847–0.855)	-
ICU admission	0.851 (0.848–0.853)	0.846 (0.843–0.848)	-
Septic shock	0.903 (0.892–0.915)	0.878 (0.860–0.897)	-
Transfer learning (5% of full data)			
Vasopressor use	0.882 (0.8742-0.890)	0.885 (0.876–0.894)	-
Respiratory support	0.897 (0.894-0.900)	0.870 (0.866–0.874)	-
ICU admission	0.863 (0.861–0.865)	0.855 (0.852–0.858)	-
Septic shock	0.901 (0.895–0.908)	0.911 (0.898–0.922)	-

*TEWS* transformer-based early warning score, *MEWS* modified early warning score, *AUROC* area under the receiver operating characteristic curve, *CI* confidence interval, *ICU* intensive care unit.

*P < 0.05 for all comparisons with MEWS.

### Model integration for EHR systems

**Fig. 3:**
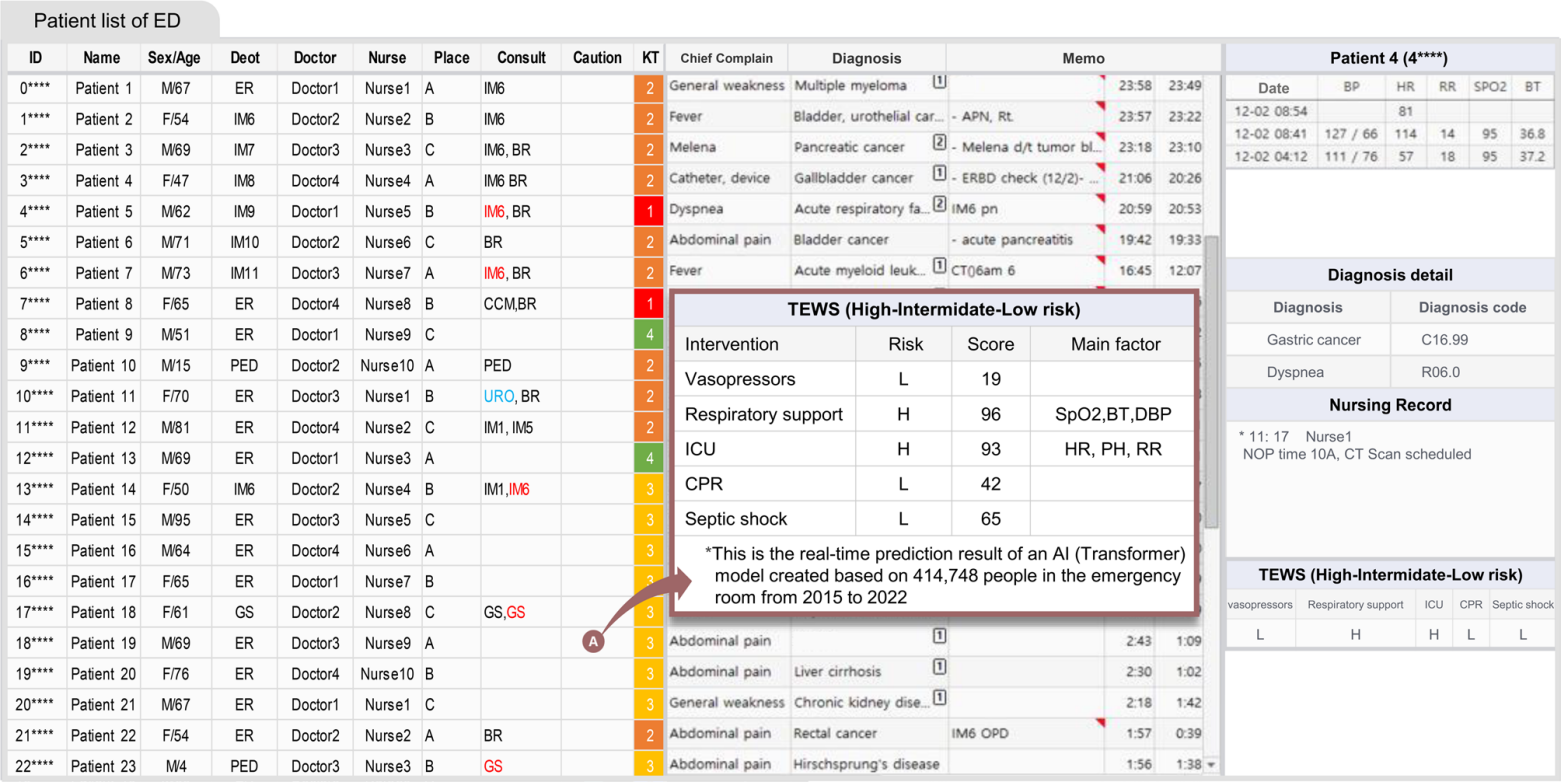
Electronic health record system integration view. This is a modified figure based on the original electronic health record system screen.

The TEWS system has been successfully integrated into the EHR system, providing real-time risk assessment for ED patients (Fig. 3). The interface displays a patient list with TEWS results that are continuously updated. When clinicians click on the TEWS alarm icon, they can access detailed information including specific high-risk outcomes and the top three contributing features identified by the AI model. When clinicians click on a patient from the ED patient list view, the right panel displays the most recent TEWS information alongside the vital signs and nursing records.

## Discussion

We developed and validated a novel early warning system for predicting adverse outcomes in ED patients, using transformer models to process time series information, including vital signs and laboratory results, from a patient’s initial visit to discharge. The TEWS system demonstrated superior prognostic performance compared to the MEWS. The TEWS includes multiple models that predict diverse outcomes, providing comprehensive information about patient deterioration from various perspectives. The TEWS predicts both procedural needs (e.g., respiratory support and vasopressor use) and patient status (e.g., cardiac arrest, ICU admission, and septic shock). This may allow TEWS to provide predictions not only about patient conditions, but also about the procedures that may be required, ultimately delivering targeted information to improve patient outcomes^13^.

While implementing AI-based systems in healthcare is complex and faces barriers such as alert fatigue and workflow integration, real-time early warning systems like TEWS can support physicians and nurses by helping prioritize patients and enabling earlier identification of those at risk for deterioration. Rather than replacing clinical judgment, TEWS may enhance situational awareness by continuously analyzing patient data and providing interpretable, outcome-specific risk predictions at the point of care. At our institution, TEWS has been incorporated into quality improvement initiatives to reduce time to blood pressure stabilization in critically ill patients and to expedite antibiotic administration in septic shock, demonstrating its potential to facilitate timely interventions. Nonetheless, sustained interdisciplinary collaboration and ongoing refinement are essential to ensure clinical value and successful adoption.

To achieve practical applicability, it is crucial to demonstrate the effectiveness of the model across datasets. AI-based models often perform well in a developmental environment, but a decrease in function may occur when they are applied externally^33^. TEWS showed acceptable predictability for most outcomes in the MIMIC-IV-ED dataset, which differs significantly from the original study site. However, we observed initial performance variations, particularly in the full TEWS model, with some outcomes showing decreased AUROC. This decline in performance often is observed in external validation and can be attributed to various factors including potential overfitting of the initial model, differences in variable distributions and measurement frequencies between institutions, variations in clinical practice patterns, and differences in patient populations^34^. Notably, the vital sign-only TEWS with limited variables demonstrated more robust performance in external validation, suggesting that models with fewer, standardized variables may be more generalizable across healthcare settings. Furthermore, the implementation of transfer learning with just 1–5% of external data significantly improved the performance of the full model, indicating that this approach could address institutional differences while requiring minimal additional data for model adaptation.

While the transformer model did not demonstrate overwhelmingly superior performance compared to XGBoost, we selected the transformer approach for its greater extensibility and practical advantages in clinical deployment. Transformer models are specifically designed to process sequential time-series data and support transfer learning, as shown by notable improvements in external validation after fine-tuning with only 1–5% of external data, a capability not feasible with tree-based models like XGBoost. Furthermore, transformer architectures allow for future integration of multimodal clinical data, such as imaging and clinical narratives, enhancing adaptability and interpretability for dynamic, real-time risk prediction in the ED. Thus, despite only modest gains in AUROC, we believe the transformer model provides a more robust and flexible platform for ongoing clinical application. The strength of the transformer model lies in its ability to accurately capture the state of variables over time, improving the accuracy of acute deterioration prediction by effectively learning temporal dependencies^17,20,35^. In the ED, the patient condition can change within a relatively short time frame; the TEWS can capture these characteristics and provide timely predictions.

We observed a relatively lower performance in predicting ICU admission and cardiac arrest compared to other outcomes. The lower predictive accuracy for ICU admission probably reflects the complex nature of ICU admission decisions in different healthcare settings. Factors beyond clinical severity, such as ED crowding and low ICU capacity, may influence admission patterns^36,37^. For example, a previous study has shown that even patients with septic shock were managed in the EDs of Korea without ICU admission^38^. Additionally, there might be differences in ICU admission criteria due to the varying characteristics among centers. This should be considered when applying TEWS externally or when developing similar models^39,40^. For cardiac arrest prediction, the extremely low incidence of in-hospital cardiac arrest in the ED presented a significant challenge for model training, and some cardiac arrests could not be predicted by vital signs and laboratory tests.

For real-world application, TEWS aims to extract essential input variables from the numerous data points available in the ED. As explained earlier, this study conducted several rounds of sensitivity analysis and feature selection to identify the most critical variables for predicting outcomes. Through this process, vital signs and laboratory variables were selected, resulting in a practical model that can be implemented in a real ED setting, reducing computing time and effort during operation.

This study demonstrates that development is only the beginning; the ultimate goal is integrating the developed model for real-time risk assessment and actionable information to healthcare providers. Through these efforts, TEWS has been integrated into the EMR system at the study site to allow monitoring and user feedback.

Further studies are needed to continue the development and enhancement of TEWS, as well as to evaluate its practical utility in clinical settings.

### Limitations

There are some limitations in our study. First, the retrospective nature may have introduced selection bias, potentially affecting the generalizability of our findings. In addition, there may be unmeasured variables or outcomes that could potentially impact the performance or generalizability of the model. Second, the incidence of the five outcomes predicted by TEWS was imbalanced. As a result, all predicted outcomes had a low AUPRC and low PPV. In the medical field, most candidates have a low likelihood of adverse outcomes because such outcomes typically occur in unhealthy states. Given that this study was based on real-world data, the imbalance is a potential limitation. Similar challenges have been reported in other machine learning-based prediction studies in healthcare^13,41,42^. Third, although we used the MIMIC-IV-ED database for external validation, this dataset may not fully represent the diversity of EDs globally. The performance of TEWS may vary by healthcare system and patient population. Fourth, there was the only moderate performance improvement after transfer learning. We believe that the moderate gains observed after transfer learning reflect a good baseline performance, and even a 2–3% improvement in AUROC can be meaningful given the limited amount of fine-tuning data used. Fifth, we also used separate models for each adverse event, which may limit the benefits of shared learning across related outcomes. A multi-task model that predicts multiple outcomes simultaneously could improve performance, especially for rare events, and will be considered in future work. Finally, the TEWS model may be challenging to implement in resource-limited settings without comprehensive EHR systems. Our model relies on time series data, which may not be consistently available or accurately recorded in all clinical settings, potentially limiting their applicability. These limitations are common challenges in developing and implementing AI-based CDSS in healthcare. Future research should focus on addressing these limitations through prospective studies, more diverse external validation, and continued refinement of the model to balance complexity with clinical applicability.

## Conclusion

This study developed and validated the TEWS system for predicting multiple adverse outcomes in ED patients. The TEWS models demonstrated superior prognostic performance compared to the MEWS across various outcomes in internal and external validation. The successful integration of the AI solution into the EHR system demonstrates its potential as a clinical decision support tool for providing real-time risk assessment and actionable information.

## Electronic supplementary material

Below is the link to the electronic supplementary material.

Supplementary Material 1

## Data Availability

Data were obtained from the study site clinical data warehouse. The datasets generated and analyzed during the current study are not publicly available because they include patient information, although it is de-identified. However, the data are available from the corresponding author on reasonable request. The external validation data used in the study are available from the Medical Information Mart for Intensive Care (MIMIC)-IV database. This third-party data can be accessed at https://mimic.physionet.org/. Access to the files requires registration as a credentialed user, completing the CITI Data or specimens-only research training, and signing the data use agreement for the project.
