# Supplementary material for: Development and validation of a transformer model-based early warning score for real-time prediction of adverse outcomes in the emergency department
Source: Sci Rep. 2025 Jul 2;15:23021. doi: 10.1038/s41598-025-07511-7 (PMC12217874; doi:10.1038/s41598-025-07511-7)
Supplement: Supplementary file 1 — Supplementary Material 1 [file 41598_2025_7511_MOESM1_ESM.docx]

**Table S1. List of candidate variables**

| **Vital signs (Acceptable range)** | **Laboratory tests** | **Other variables** |
| --- | --- | --- |
| Systolic Blood Pressure (0-350 mmHg) | White blood cell count | PaO_2_/FiO_2_ ratio |
| Diastolic Blood Pressure (0-180 mmHg) | Hemoglobin | Systolic shock index |
| Mean arterial Pressure (0-250 mmHg) | Hematocrit | Diastolic shock index |
| Peripheral oxygen saturation (0-100%) | Platelet count | ROX index |
| Heart Rate (0-299 /min) | Absolute neutrophil count | Alert/Voice/Pain/Unresponsive |
| Respiratory Rate (0-100 /min) | Absolute lymphocyte count | |
| Body Temperature (23-43 °C) | Band neutrophil | |
|  | Segmented neutrophil | |
|  | Aspartate Aminotransferase (AST) | |
|  | Alanine Aminotransferase (ALT) | |
|  | Total Bilirubin |  |
|  | Serum albumin | |
|  | Blood Urea Nitrogen (BUN) | |
|  | Serum creatinine | |
|  | Sodium |  |
|  | Potassium |  |
|  | Chloride |  |
|  | Ionized calcium | |
|  | C-Reactive Protein (CRP) | |
|  | Procalcitonin |  |
|  | Arterial pH |  |
|  | Bicarbonate |  |
|  | PaCO_2_ |  |
|  | PaO_2_ |  |
|  | Base Excess (BE) | |
|  | Lactic acid |  |
|  | Total CO_2_ (venous) | |
|  | N-Terminal pro-Brain Natriuretic Peptide  (NT-proBNP) | |
|  | Troponin T |  |
|  | D-dimer |  |
|  | Glucose |  |
|  | Prothrombin time | |

**Table S2. Baseline characteristics of the MIMIC-IV ED validation population**

| **Variables** | **At least one AE  (n = 27,838)** | **Without AE**  **(n = 383,042)** | ***P-value*** |
| --- | --- | --- | --- |
| Age, mean ± SD | 61.9 ± 18.1 | 50.1 ± 19.8 | <0.001 |
| Sex, n (%) |  |  | <0.001 |
| Male | 15,047 (45.9%) | 173,754 (45.4%) |  |
| Female | 12,791 (54.1%) | 209,288 (54.6%) |  |
| Vital sign (the first measurement) |  |  |  |
| SBP (mmHg), mean ± SD | 127.7 ± 28.8 | 132.8 ± 21.9 | <0.001 |
| DBP (mmHg), mean ± SD | 72.4 ± 18.2 | 76.7 ± 14.4 | <0.001 |
| HR (beats/min), mean ± SD | 91.8 ± 23.3 | 82.1 ± 17.0 | <0.001 |
| RR (breaths/min), mean ± SD | 19.7 ± 5.3 | 17.4 ± 2.3 | <0.001 |
| Body Temperature (°C), mean ± SD | 36.9 ± 0.8 | 36.7 ± 0.5 | <0.001 |
| SpO_2_ (%), mean ± SD | 97.0 ± 4.5 | 98.3 ± 2.6 | <0.001 |
| Laboratory tests (the first measurement) |  |  |  |
| Hb (g/dl), mean ± SD | 11.7 ± 2.6 | 12.5 ± 2.1 | <0.001 |
| BUN (mg/dl), mean ± SD | 28.7 ± 24.4 | 18.7 ± 13.9 | <0.001 |
| Sodium (mmol/L), mean ± SD | 137.5 ± 6.2 | 138.5 ± 3.8 | <0.001 |
| Potassium (mmol/L), mean ± SD | 4.5 ± 1.1 | 4.3 ± 0.8 | <0.001 |
| Lactate (mmol/L), mean ± SD | 2.8 ± 2.3 | 1.7 ± 0.9 | <0.001 |
| Arterial pH, mean ± SD | 7.3 ± 0.1 | 7.4 ± 0.1 | <0.001 |
| Arterial HCO_3_ (mmol/L), mean ± SD | 22.5 ± 5.5 | 24.5 ± 3.4 | <0.001 |
| MEWS (the first measurement), mean ± SD | 1.8 ± 1.3 | 1.2 ± 0.8 | <0.001 |
| Emergency Severity index, n (%) |  |  | <0.001 |
| 1 | 8,431 (34.2%) | 15,202 (4.0%) |  |
| 2 | 13,235 (53.7%) | 124,383 (32.7%) |  |
| 3 | 2,972 (12.1%) | 214,218 (56.3%) |  |
| 4 | 17 (0.1%) | 25,596 (6.7%) |  |
| 5 | 0 (0.0%) | 851 (0.2%) |  |
| ED disposition, n (%) |  |  | <0.001 |
| Discharge | 390 (1.4%) | 232,111 (60.6%) |  |
| ED death | 92 (0.3%) | 110 (0.0%) |  |
| Admission | 26,990 (97.0%) | 130,314 (34.0%) |  |
| Transfer or others | 366 (1.3%) | 20,507 (5.4%) |  |
| Adverse events, n (%) |  |  | <0.001 |
| Vasopressor use | 3,419 (12.3%) | - |  |
| Respiratory supports | 8,257 (29.7%) | - |  |
| ICU admission | 26,796 (96.3%) | - |  |
| Septic shock | 1141 (4.1%) | - |  |

AE, Adverse Event; SD, Standard Deviation; SBP, Systolic Blood Pressure; DBP, Diastolic Blood Pressure; HR, Heart Rate; RR, Respiratory Rate; SpO_2_, Peripheral Oxygen Saturation; Hb, Hemoglobin; BUN, Blood Urea Nitrogen; MEWS, Modified Early Warning Score; ED, Emergency Department; ICU, Intensive Care Unit

**Figure S1. MIMIC-IV-ED validation population**

**
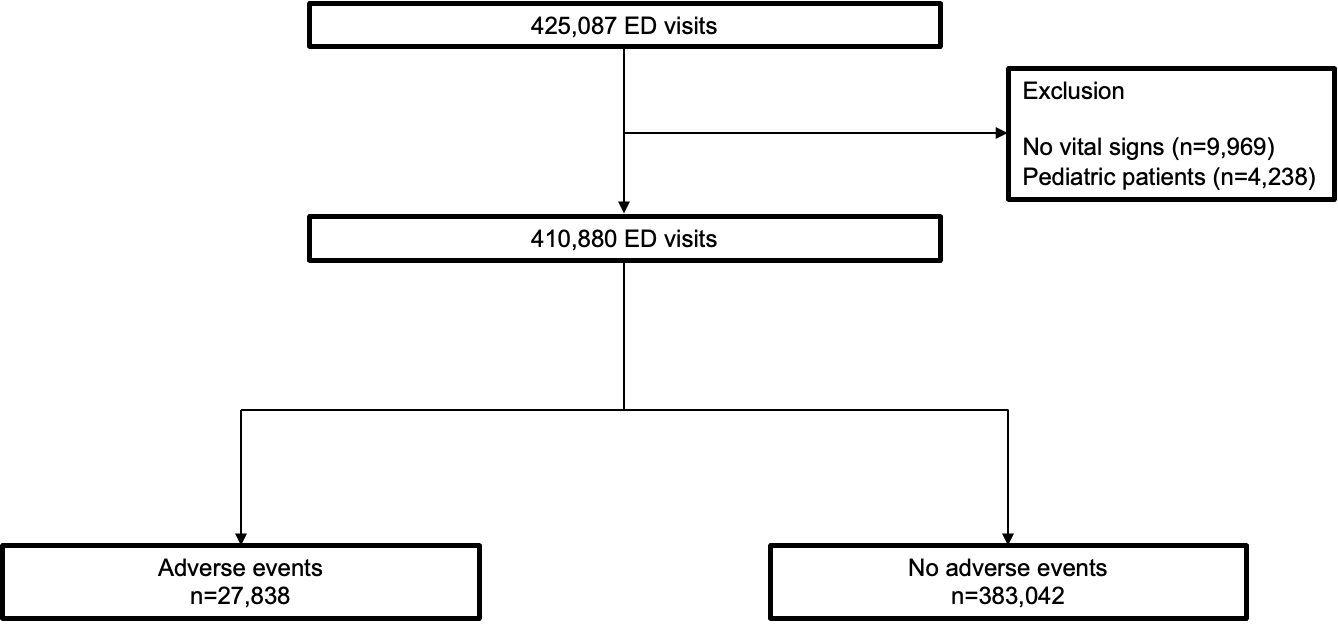
**

**Figure S2. Initial analyses for feature importance using 44 variables. The graph shows the predictive performance contribution of variables as measured by AUROC (X-axis). A. Vasopressor use; B. Respiratory support; C. Intensive care unit admission; D. Cardiac arrest**

**A B**


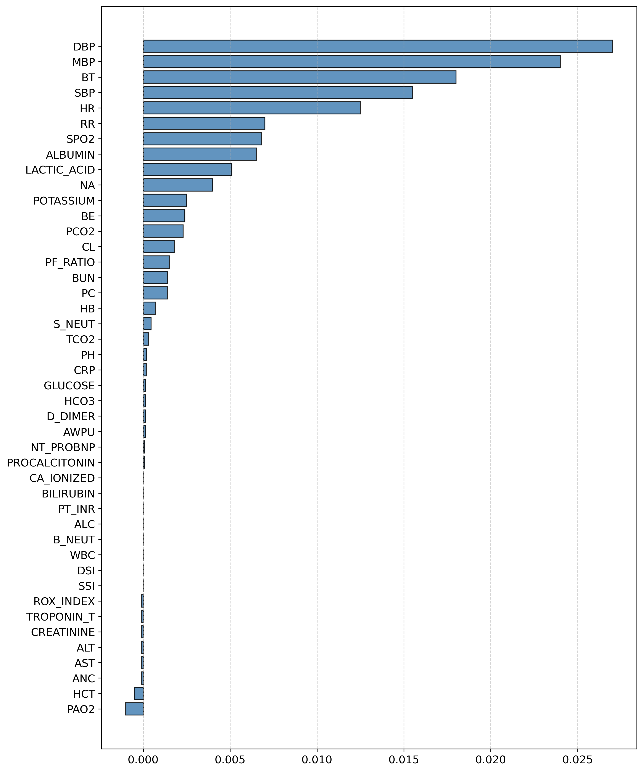

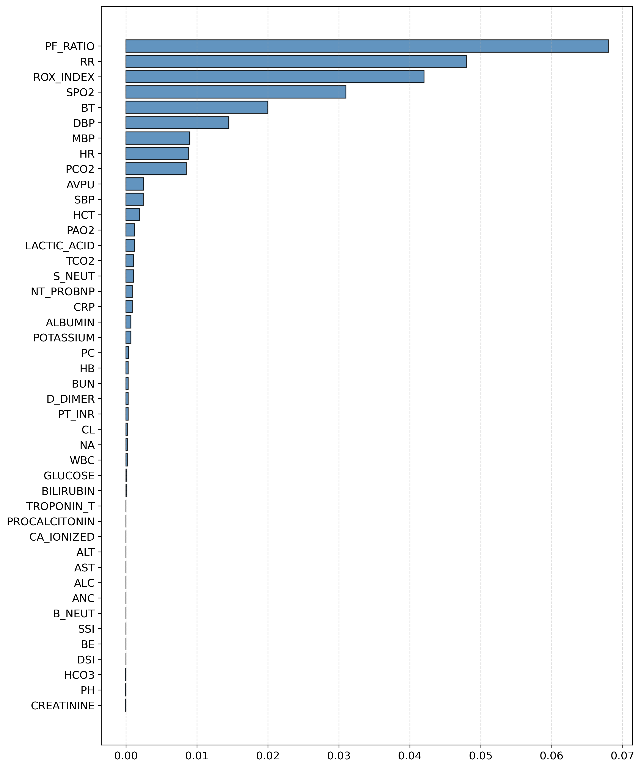


**C D**


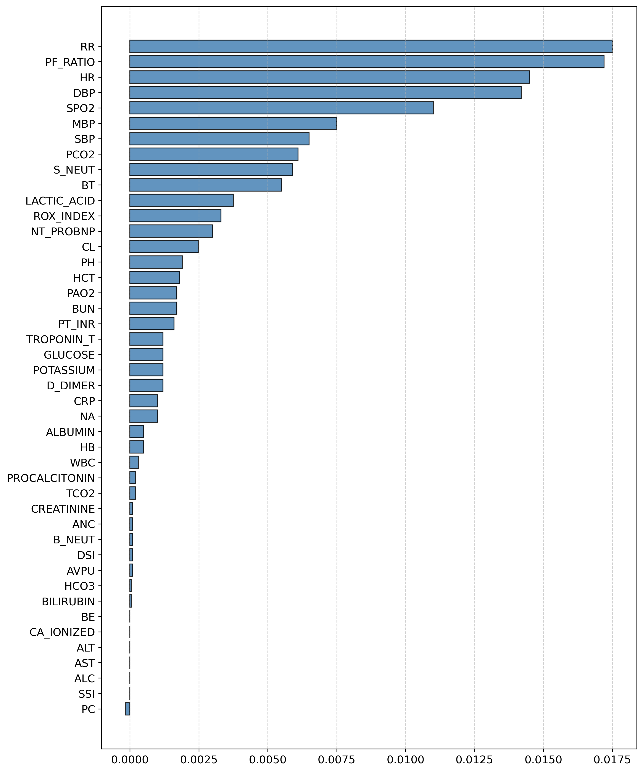

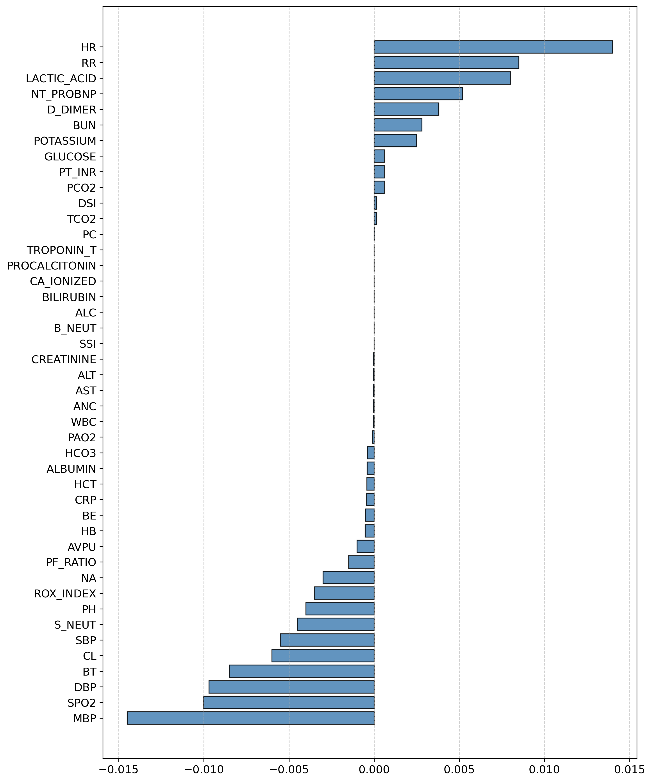


**Table S3. Prognostic performance of the TEWS models for predicting the adverse outcomes**

|  | **AUPRC (CI)** | **Sen (95% CI)** | **Spec (95% CI)** | **PPV (95% CI)** | **NPV (95% CI)** |
| --- | --- | --- | --- | --- | --- |
| Vasopressor use | 0.380  (0.371-0.389) | 0.866  (0.860-0.872) | 0.867  (0.866-0.868) | 0.135  (0.133-0.137) | 0.996  (0.996-0.996) |
| Respiratory support | 0.163  (0.156-0.171) | 0.848  (0.839-0.856) | 0.832  (0.831-0.833) | 0.064  (0.062-0.066) | 0.998  (0.997-0.998) |
| ICU admission | 0.236  (0.232-0.240) | 0.786  (0.784-0.789) | 0.773  (0.770-0.776) | 0.115  (0.113-0.117) | 0.990  (0.989-0.990) |
| Septic shock | 0.271  (0.261-0.282) | 0.872  (0.864-0.879) | 0.860  (0.859-0.861) | 0.083  (0.081-0.085) | 0.998  (0.998-0.998) |
| Cardiac arrest | 0.017  (0.013-0.023) | 0.744  (0.715-0.774) | 0.777  (0.776-0.779) | 0.005  (0.005-0.005) | 1.000  (0.999-1.000) |

*The threshold for the model was determined based on the Youden Index to maximize the trade-off between sensitivity and specificity

**Table S4. Prognostic performance of the TEWS models according to the number of variables (23 and 44 variable model)**

|  | **AUROC (95% CI),**  **(23 variables)** | **AUROC (95% CI CI),**  **(44 variables)** |
| --- | --- | --- |
| Vasopressor use | 0.925 (0.923 – 0.927) | 0.932 (0.930 – 0.933) |
| Respiratory support | 0.929 (0.927 – 0.931) | 0.920 (0.918 – 0.922) |
| ICU admission | 0.862 (0.860 – 0.864) | 0.853 (0.851 – 0.855) |
| Septic shock | 0.935 (0.933 – 0.937) | Not tested |
| Cardiac arrest | 0.851 (0.850 – 0.852) | 0.861 (0.851 – 0.870) |

TEWS, Transformer-based Early Warning Score; AUROC, Area Under the Receiver Operating Characteristic curve; CI, confidence interval; ICU, Intensive Care Unit

**Table S5. Area under the receiver operating characteristic of other models for predicting the adverse outcomes**

|  | **Logistic regression**  **(AUROC, 95% CI)** | **XgBoost**  **(AUROC, 95% CI)** |
| --- | --- | --- |
| Vasopressor use | 0.886 (0.883-0.889)^*^ | 0.932 (0.930-0.935) |
| Respiratory support | 0.856 (0.851-0.861)^*^ | 0.906 (0.903-0.910)^*^ |
| ICU admission | 0.829 (0.825-0.832)^*^ | 0.849 (0.846-0.852)^*^ |
| Septic shock | 0.882 (0.879-0.886)^*^ | 0.933 (0.930-0.935) |
| Cardiac arrest | 0.768 (0.748-0.788)^*^ | 0.826 (0.810-0.841) |

AUROC, Area Under the Receiver Operating Characteristic curve; CI, confidence interval; ICU, Intensive Care Unit

*p-value < 0.05 for comparisons with the TEWS model
